# The annual costs of cardiovascular diseases and mental disorders attributable to job strain in France

**DOI:** 10.1186/1471-2458-13-748

**Published:** 2013-08-13

**Authors:** Hélène Sultan-Taïeb, Jean-François Chastang, Malika Mansouri, Isabelle Niedhammer

**Affiliations:** 1 Département d’organisation et ressources humaines, Université du Québec à Montréal (UQAM), Montréal, Québec, Canada; 2 Centre de recherche interdisciplinaire sur la biologie, la santé, la société et l’environnement (CINBIOSE), Montréal, Québec, Canada; 3 Laboratoire d’Économie Gestion (UMR CNRS 5118), Université de Bourgogne, Dijon, France; 4 INSERM, U1018, CESP Centre for research in epidemiology and population health, Epidemiology of occupational and social determinants of health team, Villejuif, France; 5 Univ Paris-Sud, UMRS 1018, Villejuif, France; 6 Université de Versailles St-Quentin, UMRS 1018, Villejuif, France

**Keywords:** Work stress, occupational stress, job stress, job strain, attributable fraction, cost, economic burden

## Abstract

**Background:**

Work stress has become a major occupational risk factor in industrialized countries and an important economic issue. The objective was to estimate the annual costs of coronary heart diseases (CHD) and mental disorders (MD) attributable to job strain exposure according to Karasek’s model in France for the year 2003 from a societal perspective.

**Methods:**

We produced attributable fraction estimates which were applied to the number of cases (morbidity and mortality) and the costs of CHD and MD. Relative risk estimates came from a systematic literature review of prospective studies. We conducted meta-analyses based on this selection of studies. Prevalence of exposure to job strain came from the national SUMER survey conducted in France in 2003. Costs included direct medical costs and indirect costs: production losses due to sick leaves and premature deaths.

**Results:**

Between 8.8 and 10.2% of CHD morbidity was attributable to job strain, and between 9.4 and 11.2% of CHD mortality was attributable to this exposure for men. Between 15.2 and 19.8% of MD was attributable to job strain for men, and between 14.3 and 27.1% for women. As a whole, between 450 000 and 590 000 cases of diseases and 910–1130 deaths were attributable to job strain for men. From 730 000 to 1 380 000 cases of diseases and from 150 to 280 deaths were attributable to job strain for women. The total number of sick leave days amounted from 5 to 6.6 million days for men, and from 8.5 to 16 million days for women. The total costs of CHD and MD attributable to job strain exposure ranged from 1.8 to 3 billion euros for the year 2003 (0.12-0.19% GDP). Medical costs accounted for 11% of the total costs, value of life costs accounted for 13-15% and sick leave costs for 74-77%. The cost of CHD was estimated at 113–133 million euros and the cost of MD was between 1.7 - 2.8 billion euros in 2003.

**Conclusion:**

This study on the economic burden of diseases attributable to job strain in France provides relevant insights for policy-makers when defining public health priorities for prevention policies.

## Background

Work stress has become a major occupational risk factor in all industrialized countries [1] and an important economic issue. The high cost of work stress for employers, social security systems and society as a whole has been emphasized in numerous studies [2-5] but detailed evaluations of these costs have been very seldom in the literature. A few rough estimates were produced without explaining the method used for such estimates. For example, costs of work stress were estimated at €20 trillion in Europe per year [6], but details on the estimation method were not available. Moreover, the discrepancy between estimates can be very large [7]. This is partly due to the fact that work stress can be defined as a risk factor (related to working conditions and work organization) or as a health outcome related to mental disorders. For example, Hoel and al [8] estimated that the costs of work stress and bullying may account for approximately 0.5-3.5% of GDP per year in 2000 in the UK (approx £4.9-34.1 billion), based on the hypothesis that these risk factors represented 30% of the overall cost of ill-health and accidents. The Health and Safety Executive in the UK estimated the cost of sickness absence due to stress, depression and anxiety perceived by the worker as being ‘caused or made worse by work’ (Labour Force Survey, UK) at £530 million in 2006 [9].

A way to evaluate the cost of work stress is to measure claims costs due to stress-related conditions at work. But this type of evaluation depends on national legislative frameworks which vary from one country to another. For example, in Australia, attempts have been made to reduce the costs of stress-related claims by modifying legislative thresholds on such claims [10]. In general, occupational exposures costs based on workers compensations only reflect a part of total costs, since occupational diseases or injuries have to be declared by workers [11-13] and covered by social welfare or insurance schemes to appear as claims [14].

Attributable fractions have been widely used in the literature to estimate the burden of diseases attributable to occupational exposures in terms of morbidity or mortality cases and disability-adjusted life years [15-20]. Attributable fraction can also be used to estimate the monetary cost of diseases attributable to occupational exposures but literature remains very sparse on this issue, except for some studies [21-23]. Studies are even less numerous when considering the costs of disease attributable to exposures to psychosocial risk factors at work [24-26]. However, precise and detailed economic evaluations of the costs associated to psychosocial work exposures are much needed to show the magnitude of the problem, and to provide guidance to policy makers when defining public health priorities and allocation of limited resources [27].

A large number of epidemiologic studies have shown that exposure to work stress is a risk factor for various health outcomes, especially cardiovascular diseases [28,29] and mental disorders [30-32]. Less conclusive results have been found for musculoskeletal diseases [33,34]. The leading work stress model in the literature is Karasek’s job strain model [35]. This model is based on two dimensions: psychological demands (i.e. job demands, time pressure, conflicting demands, etc.), and decision latitude (i.e. both control over work, named decision authority, and possibility of developing skills, named skill discretion). According to this model, the most detrimental situation is defined by the combination of high levels of psychological demands and low levels of decision latitude, and is called *job strain*. Data on the impact of job strain on workers’ health are available in the literature and exposure to job strain has been measured in a national survey, representative of the working population, in France in 2003.

The aim of this article was to estimate the costs of cardiovascular diseases (CVD), and more precisely coronary heart diseases (CHD) and mental disorders (MD), i.e. two highly prevalent common mental disorders, depression and anxiety, attributable to job strain exposure in France in 2003 from a societal perspective, using a cost-of-illness approach [36,37]. A societal perspective is used to take account of all the costs associated with a disease, no matter who bears the costs, including health services and insurances, patients and the production system (lost production). This approach is considered as appropriate for decision making concerning public health policies [38-40].

## Methods

### AF data and calculation

As defined by Nurminen and Karjalainen [19], attributable fractions (AF) are an estimate of the fraction of cases that is “attributable to an exposure in a population and that would not have been observed if the exposure had been non-existent”. AF calculations are based on relative risk *RR* estimates (risks of disease or death due to exposure to the risk factor) and prevalence of exposure *P*_*e*_ estimates (proportion of the population exposed to the risk factor) [41]:

(1) $$\mathrm{AF}=P_{e}\left(RR-1\right)/\left(1+P_{e}\left(RR-1\right)\right)$$

RR estimates were derived from a systematic literature review of prospective studies published in peer-reviewed journals between 1998 and 2008 (inclusive), i.e. a period centered around the year 2003. Inclusion criteria were a sufficient sample size (over 100 individuals) of a population in industrialized countries, with an explicit use of Karasek’s model for exposure assessment and a precise measurement of health outcomes (clinically diagnosed or based on validated instruments). Only studies studying men and women separately and providing unadjusted (or age-adjusted) and multi-adjusted RRs/ORs were retained. This literature review excluded studies based on a job-exposure matrix known to underestimate RRs [42]. Fifteen prospective studies were selected, more details on the selection process of the literature review are available elsewhere [34].

In order to summarize the results of this literature review, we conducted meta-analyses based on this selection of studies. We retained 9 studies for CVD [43-51] among which 8 produced RRs for CHD and 1 study yielded RR estimates for CVD [46], i.e., for a broader range of cardiovascular outcomes. We included 6 studies for MD [52-57]. Depression and anxiety were evaluated using either validated self-administered questionnaires, such as the Beck Depression Inventory (BDI), Center for Epidemiologic Studies Depression Scale (CES-D), General Health Questionnaire (GHQ), and Psychiatric Symptom Index (PSI), or standardized diagnostic interviews such as CIDI (Composite International Diagnostic Interview). We calculated two sets of summary RR estimates separately based (1) on unadjusted or age-adjusted RRs and (2) on multi-adjusted RRs. We produced summary estimates for men and women separately. We used a random effects method with inverse variance weighting, which is considered as more conservative than the fixed effects method [28,30]. Two indicators of heterogeneity between studies were used: Cochran’s Q statistic with a *p* value <0.05 for a significant heterogeneity and the I^2^ statistic [58]. We used Stata software for meta-analysis calculations.

The data used for the estimate of prevalence of exposure P_e_ to job strain came from the national SUMER survey which was conducted in France in 2003. This survey is a periodical cross-sectional survey conducted by the French Ministry of Labour (DARES) and including a self-administered questionnaire, including Karasek’s Job Content Questionnaire (JCQ). In total, 24,486 employees, 12,241 men and 10,245 women, selected on a random basis responded to the JCQ (response rate: 96.5%). The data of the SUMER survey were weighted to provide estimates that were representative of the French working population. As a result, the 2003 SUMER survey provides high quality data for exposure to job strain among the French national working population according to gender [42,59-62]. The estimates for the prevalence of job strain exposure obtained from the SUMER survey were 19.6% for men, 28.2% for women, and 23.2% for the total population.

We produced a range of attributable fraction (AF) values. The AF low range value for each disease was calculated using multi-adjusted RR summary estimates in formula (1) and the AF high range value was based on age-adjusted RR summary estimates. In this calculation, we assumed that ORs were satisfactory estimates for RRs, which allowed to use ORs for AFs calculation in formula (1).

We computed confidence intervals at 95%, based on 100 000 simulated distributions of *P*_*e*_ and *RR*, with the hypothesis that *RR* and *P*_*e*_ follow independent normal distributions. Confidence intervals were calculated with the mean and standard deviation from the simulated values of AFs. We used SAS software for simulation calculations.

### Data on prevalence of diseases

We considered all national surveys available in France for the number of cases (morbidity and mortality) of CVD and MD. Inclusion criteria were a definition of disease corresponding to health outcomes used in our summary RR estimates, the availability of data for men and women separately, the availability of data for working-age population, and the survey had to be conducted in 2003 or as close as possible to 2003.

#### CHD morbidity data

Since most of the selected studies for CVD produced RRs for CHD (8 studies out of 9), we retained prevalence and costs data on CHD rather than CVD to ensure consistency between data used in our estimation. We used the prevalence of CHD (CIM 10, codes I20-I25) from the database of the Health and Social Benefits Survey (Enquête Santé et Protection Sociale) conducted in France in 2004 by IRDES (Institut de Recherche et Documentation en Économie de la Santé). Weighted prevalence data were computed and provided by IRDES for men (1.38%) and women (0.37%) from 18 to 64 years. We assumed a stable prevalence of CHD between 2003 and 2004 in France.

#### CHD mortality data

We used mortality data in 2003 from the database of the Epidemiological Center for the Medical Causes of Death (Centre d’Épidémiologie sur les Causes Médicales de Décès, CEPIDC) [63]. It provides the number of deaths from CHD (CIM 10, codes I20-I25) for men and women from 20 to 64 years.

#### MD morbidity data

We computed the prevalence of MD (depression and anxiety) from a French national survey, the Ten-yearly Health survey 2002–2003 (Enquête Décennale Santé). Data were weighted by age and gender. MD were defined by a score obtained to CES-D equal or higher than 16. The prevalence of MD for the population from 18 to 64 years was 15.5% (95% confidence interval: 14.6%-16.4%) for men and 27.4% (95% confidence interval: 26.4%- 28.4%) for women in France.

#### MD mortality data

We used data about the prevalence of suicide in 2003 provided by the Epidemiological Center for the Medical Causes of Death (Centre d’Épidémiologie sur les Causes Médicales de Décès, CEPIDC) for men and women from 20 to 64 years [63]. Based on an analysis of the literature [64-66], we assumed that from 54 to 64% of suicides were due to depression. A sensitivity analysis was conducted to take account of these two percentages.

Another sensitivity analysis was conducted using a more restrictive definition of mental disorders based on depression alone. For this analysis, we used depression morbidity data produced by ESEMED study (European Study of the Epidemiology of Mental disorders) which was based on a standardized diagnostic interview (WMH-CIDI: World Mental Health- Composite International Diagnostic Interview). According to this survey, the prevalence of depression was 4.7% (95% confidence interval: 3.6%-5.8%) for men and 8.5% (95% confidence interval: 7.1%-9.9%) for women in France [67]. We computed AF estimates for depression using job strain RR estimates provided by Shields’s [57] study. It was the only study in our literature review based on a measure of depression using a standardized diagnostic interview (CIDI) and producing both crude and multi-adjusted RR estimates.

### Cost data and calculation

Costs *C* were estimated from a societal perspective according to a prevalence approach and based on French data. They included medical costs *M* of CHD and MD and indirect costs: production losses due to sick leaves *S* and to premature deaths *D* attributable to job strain exposure, according to the following formula:

(2) $$C=\sum_{i}^{\mathrm{CVD},\mathrm{MD}}\left(M_{i}+S_{i}+D_{i}\right)AF_{i}$$

where *AF*_*i*_ is the attributable fraction corresponding to disease *i* (CHD or MD). Data on production losses due to early retirement and to presenteeism were not available. For the data on medical and indirect costs of diseases we conducted a systematic review of French and international databases, reports (EUROHEED-CODECS, DREES, IRDES, CNAMTS, Base BDSP, OECD, WHO) and articles published in peer reviewed journals through Medline interrogations.

Indirect costs were calculated on the basis of the human capital hypothesis [68], by multiplying the number of lost days because of illness with the Gross Domestic Product per capita and per working day (*GDP*_*cd*_) for the year 2003 in France. The value of production losses due to premature death is estimated to take account of the lost production due to the number of years lost between the age of death and the average retirement age, as shown in formula (3):

(3) $$\mathrm{VLY}=\mathrm{GD}P_{\mathrm{cd}}\sum_{a}\left(N_{a}\sum_{y=1}^{R-a}\frac{{\left(1+g\right)}^{y}}{{\left(1+r\right)}^{y}}\right)$$

Where *VLY* is the value of lost years of production due to disease (CHD or MD), *GDP*_*cd*_ is the French Gross Domestic Product per capita and per working day, *N*_*a*_ is the number of deaths due to the disease at the age *a* in the French population, *R-a* is the number of years lost between the age of death *a* and the average retirement age *R*, *g* is the annual growth rate of GDP and *r* the discount rate. A discount rate of 5% and a growth rate of 2% were posited, as generally assumed.

A sensitivity analysis was conducted to take account of the two range values for the number of suicides due to depression in the literature (54% or 64%) and the two values of attributable fractions which we computed (from multi or age-adjusted RRs).

#### CHD medical costs

According to Paris and al [69], CHD represented 16% of national consumption of medical care and goods for diseases of the circulatory system in France in 1998. And according to Fenina and al [70], diseases of the circulatory system represented 12.6% of the total consumption of medical care and goods in France in 2002. We assumed that average medical expenses for each case of CHD were the same between genders and age groups and a stable proportion of CHD medical expenses in total consumption of medical care and goods from 1998 to 2003 in France.

#### CHD indirect costs

Statistics from the public medical care system provided the number of sick leave days as prescribed by cardiologists in France in 2003 [71]. We made the assumption that the distribution of sick leave days between men and women was similar to the distribution of CHD cases between genders.

#### MD medical costs

According to Paris and al [69], depression represented 1.05% of the total consumption of medical care and goods in France in 1998. We assumed that medical expenses for each case of MD were the same between genders and age groups and a stable proportion of MD medical expenses in total consumption of medical care and goods from 1998 to 2003.

#### MD indirect costs

A study by Morvan et al. [72], on the basis of a national survey conducted in France in 2005 (Baromètre Santé 2005), produced the proportion of mild, average and severe episodes of depression in the population suffering from depression and the corresponding number of sick leave days per year. According to this survey, 17.8% of persons suffering from mild depressive episodes take an average of 10.4 sick leave days per year. 26.2% of persons suffering from average depressive episodes take an average of 49.2 sick leave days per year. And 48.9% of persons suffering from major depressive episodes take an average of 108.1 sick leave days per year. We assumed that the distribution of sick leave days between men and women was similar to the distribution of depression cases between genders. We also assumed that the number of sick leave days was stable between 2003 and 2005. Health reasons for sick leaves are not available in health insurance databases in France because the medical cause of absence is covered by confidentiality.

## Results

**Figure 1:**
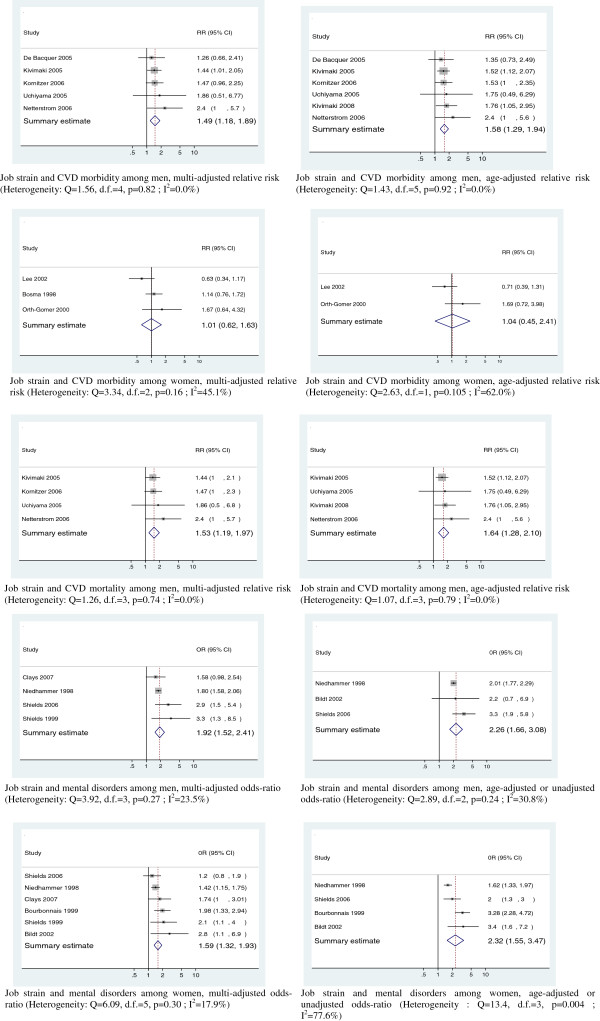
Meta-analysis.

Meta-analysis results are shown in Figure 1. Summary RR estimates for CHD morbidity were significant for men, 1.49 and 1.58, and not significant for women, 1.01 and 1.04. Significant summary RRs for CHD mortality were 1.53 and 1.64 for men. There was no multi-adjusted RR for CHD mortality among women and only one non-significant age-adjusted RR estimate. Thus available data did not allow the calculation of summary RR estimates for CHD mortality among women. Summary OR estimates for MD were significant for both genders, 1.92 and 2.26 for men, 1.59 and 2.32 for women. Summary RR and OR estimates and 95% confidence intervals are shown in Table 1.

**Table 1:** Summary relative risks (RR) and odds-ratios (OR) on the association between job strain and coronary heart diseases (CHD) and mental disorders (MD)

		**CHD**						**MD**		
		**Morbidity RR**	**95% CI**		**Mortality RR**	**95% CI**		**Morbidity OR**	**95% CI**	
Men	M-A	1.49	1.18	1.89	1.53	1.19	1.98	1.92	1.52	2.41
	A-A	1.58	1.29	1.94	1.64	1.28	2.10	2.26	1.66	3.08
Women	M-A	1.01	0.62	1.63	/			1.59	1.32	1.93
	A-A	1.04	0.45	2.41	1.09*	0.4	2.92	2.32	1.55	3.47

M-A: multi-adjusted, A-A: age-adjusted or unadjusted.

* Estimate from one study only (Lee et al., [48]), not a summary RR.

95% CI : confidence interval at 95%.

For men, 8.8-10.2% of CHD morbidity was attributable to job strain. Attributable fractions (AF) for CHD mortality were 9.4-11.2% for men. AFs for CHD morbidity and mortality for women yielded non significant values (Table 2). Therefore, we could not infer the burden of CHD attributable to job strain exposure for women, for lack of significant RR data. Between 15.2 and 19.8% of MD were attributable to job strain for men, and between 14.3 and 27.1% for women, these two AFs being significant. AF estimates and 95% confidence intervals are shown in Table 2.

**Table 2:** Fractions of coronary heart diseases (CHD) and mental disorders (MD) attributable to job strain in France (%)

		**CHD**						**MD**					
		**Morbidity**	**95% CI**		**Mortality**	**95% CI**		**Morbidity**	**95% CI**		**Mortality**	**95% CI**	
Men	M-A	8.8	3.2	14.7	9.4	3.3	15.9	15.2	9.0	21.5	15.2	9.0	21.5
	A-A	10.2	5.2	15.4	11.2	4.9	17.5	19.8	11.1	28.7	19.8	11.1	28.7
Women	M-A	0.2	−13.1	14.1	/			14.3	8.1	20.7	14.3	8.1	20.7
	A-A	1.1	−21.6	25.9	2.5	−24.7	31.9	27.1	13.2	41.0	27.1	13.2	41.0

M-A: attributable fraction based on multi-adjusted relative risk, A-A: attributable fraction based on age-adjusted or unadjusted relative risk.

95% CI: confidence interval at 95%.

Between 22 071 and 25 743 cases of CHD were attributable to job strain exposure, and between 442 and 524 deaths among men. For MD, between 430 470 and 559 215 cases for men and 727 266–1 378 718 cases for women were attributable to job strain exposure. Between 619 and 894 deaths (suicides associated with depression) for men and women were attributable to this exposure. The number of sick leave days lost because of CHD attributable to job strain went from 55 015 to 64 167 days. For MD, the number of lost working days amounted from 5 016 529 to 6 516 864 days for men and from 8 475 264 to 16 067 034 days for women (Table 3).

**Table 3:** Burden of coronary heart diseases (CHD) and mental disorders (MD) imputable to job strain exposure in France in 2003

	**Men**		**Women**		**Total**	
	**Multi-adjusted**	**Age-adjusted**	**Multi-adjusted**	**Age-adjusted**	**Multi-adjusted**	**Age-adjusted**
CHD morbidity	22 071	25 743	-	-	22 071	25 743
CHD mortality	442	524	-	-	442	524
MD morbidity	430 470	559 215	727 266	1 378 718	1 157 736	1 937 933
MD mortality	469	610	150	284	619	894
Number of sick leave days for CHD	55 015	64 167	-	-	55 015	64 167
Number of sick leave days for MD	5 016 529	6 516 864	8 475 264	16 067 034	13 491 794	22 583 898
Total number of cases	452 542	584 957	727 266	1 378 718	1 179 807	1 963 676
Total number of deaths	912	1 134	150	284	1 062	1 418
Total number of sick leave days	5 071 545	6 581 031	8 475 264	16 067 034	13 546 809	22 648 065

The total costs of CHD and MD attributable to job strain exposure ranged from approximately 1.8 to 3 billion euros per year in France. This cost included medical costs, the value of sick leave days lost because of disease, and the value of life years lost because of premature death. The cost of CHD was estimated at 113–133 million euros. The cost of MD was around 1.7 and 2.8 billion euros. Results are shown in Table 4.

**Table 4:** Costs of diseases imputable to job strain en France in 2003 (euros)

	**Men**		**Women**		**Total**	
	**Multi-adjusted**	**Age-adjusted**	**Multi-adjusted**	**Age-adjusted**	**Multi-adjusted**	**Age-adjusted**
CHD						
Medical costs	41 107 656	47 945 576			41 107 656	47 945 576
Sick leave costs	5 543 673	6 465 817			5 543 673	6 465 817
Value of life costs	66 565 007	78 813 896			66 565 007	78 813 896
Total CHD	113 216 336	133 225 290	-	-	113 216 336	133 225 290
MD						
Medical costs	60 368 981	78 424 030	101 991 441	193 350 898	162 360 422	271 774 928
Sick leave costs	505 494 140	656 676 437	854 015 999	1 619 006 057	1 359 510 139	2 275 682 494
Value of life costs	156 839 921	203 747 328	47 243 181	89 561 550	204 083 102	293 308 877
Total MD	722 703 042	938 847 795	1 003 250 621	1 901 918 505	1 725 953 663	2 840 766 300
Total	835 919 378	1 072 073 084	1 003 250 621	1 901 918 505	1 839 170 000	2 973 991 589

CHD: coronary heart diseases, MD: mental disorders.

We computed a sensitivity analysis based on a less conservative hypothesis for the percentage of suicides associated with depression, according to data available in the literature [64-66]. Assuming that 64% of suicides were associated with depression instead of 54%, the number of deaths related to MD attributable to job strain was between 556 and 723 for men, and between 178 and 337 for women. As a result, the total number of deaths increased by 18.5% and the total costs increased by around 2%. Therefore, this alternative hypothesis had a limited impact on the total costs attributable to job strain exposure.

We also conducted a more conservative analysis based on a restrictive definition of mental disorders, including depression only. RR estimates for depression were derived from Shields’s study [57] and were from 2.9 to 3.3 for men and from 1.2 to 2 for women, with a non-significant multi-adjusted RR estimate for women. Thus, available data did not allow the calculation of AF based on multi-adjusted RR estimate for women. For men, 27.2-31.1% of depression was attributable to job strain and 22% for women. Between 232 308 and 266 007 cases of depression for men and 346 172 cases of depression for women were attributable to job strain exposure. The number of lost working days because of depression attributable to job strain amounted from 2 707 220 to 7 134 095 days for men and women. The cost of depression attributable to job strain was between 780 million and 1.6 billion euros.

## Discussion

Between 8.8 and 10.2% of CHD morbidity was attributable to job strain, and between 9.4 and 11.2% of CHD mortality was attributable to this exposure for men. Between 15.2 and 19.8% of MD (depression and anxiety) was attributable to job strain for men, between 14.3 and 27.1% for women. As a whole, between 450 000 and 590 000 cases of diseases and 910–1130 deaths were attributable to job strain for men. From 730 000 to 1 380 000 cases of diseases and from 150 to 280 deaths were attributable to job strain for women. The number of deaths attributable to job strain is approximately twice the number of deaths due work fatalities declared in France in 2003. The total number of sick leave days for the year 2003 amounted from 5 to 6.6 million days for men, and from 8.5 to 16 million days for women. The total costs of CHD and MD attributable to job strain exposure ranged from approximately 1.8 to 3 billion euros for the year 2003 in France (0.12-0.19% GDP). Medical costs accounted for 11% of the total costs, value of life costs accounted for 13-15% and sick leave costs for 74-77%. The cost of CHD was estimated at 113–133 million euros and the cost of MD was between 1.7 and 2.8 billion euros. Thus MD accounted for approximately 94-96% of the total costs attributable to job strain exposure. This is due to a higher prevalence of the disease among the population of working age, higher levels of attributable fractions, and also a high amount of sick leave days due to MD. The sensitivity analysis conducted on a more restrictive definition of mental disorders including depression only, produced much lower values in terms of number of cases and costs. This calculation can be considered as very conservative. Indeed, the definition of mental disorders was restricted to clinical depression, and thus excluded anxiety but also symptomatic cases of depression and anxiety. Furthermore, the RR estimates were based on only one available prospective study that produced RR estimates using a standardized diagnostic interview (CIDI) [57]. This study yielded non-significant multi-adjusted RR estimates for women, which did not allow to calculate a low range value of AF estimates.

Some limitations of the study must be pointed out. This estimate of the cost of diseases attributable to job strain exposure may be more under-estimated than over-estimated. We did not produce AF estimates of CHD morbidity and mortality for women since available data were very scarce and the rare included studies yielded non-significant RRs. Therefore the estimation of the cost of CHD attributable to job strain was provided for men only. Another way of interpreting the results may be that our estimates of costs may be null for women, something that may be a bit premature to conclude given the scarcity of the literature. Indeed, recent studies show an increase in CVD prevalence among women in the French population [73,74], which underlines the need for more investigations on the etiological role of job strain on CVD among women. The estimates did not take account of potential delayed effects of job strain exposure on cardiovascular or mental health. We included only CHD and MD in our study. The number of studies selected in our literature review was very small for musculoskeletal disorders (MSD) and concerned different locations (back, low back, neck, shoulder, upper extremity, elbow, hand, and wrist) [34]. Therefore we could not compute a summary OR and AF estimates for a specific MSD location which could be used for cost of illness estimates. Our study grasped only a part of the total amount of costs attributable to work stress since job strain is only one aspect of stress among other concepts, such as Effort-Reward Imbalance developed by Siegrist [75]. Cost data had also some limitations. Costs did not include production losses due to presenteeism and to early retirement for lack of data. Other cost categories could not be included in our estimates, such as the costs of informal care (provided by families and friends), intangible costs (cost of suffering, pain and discomfort) and the cost of job turnover for lack of data. We also limited the value of lost years of life to the value of lost production. We did not take account of the total losses of social welfare due to deaths through an estimation of willingness to pay or value of a statistical life for instance, for lack of available data. For all these reasons, our estimates can be considered as conservative. In addition, we made a number of assumptions in our calculations and these assumptions may not be verified easily. For example, we assumed that the RR of mental disorders associated with job strain was the same for morbidity and mortality, something that may be consistent for cardiovascular diseases but was not checked for mental disorders, the literature being rare on this topic [76]. Another example, the proportion of suicides due to depression was estimated to be 54%-64% of the total number of suicides in the general population but it was not possible to check whether these figures may be used for the working population.

This study has also several strengths. Our meta-analyses for RR estimates showed a satisfactory homogeneity between studies, except for age-adjusted ORs of MD for women which yielded a significant level of heterogeneity. Overall, the risk of heterogeneity between studies was mitigated by the fact that we included only high quality studies in our systematic review of the literature, with similar exposure and with statistical analyses allowing the estimation of RRs. The data we used are highly consistent: RR, prevalence of exposure, prevalence of disease and cost data are based on the same definition of disease and exposure. To ensure such consistency, we conducted a systematic review of the literature for disease prevalence and costs data in France and we produced data for each gender separately. The only exception may be the use of mental disorders medical costs based on the medical cost of depression only that may underestimate the costs of mental disorders attributable to job strain. We performed a sensibility analysis including depression only to provide a very conservative estimate of mental disorders costs attributable to job strain. The medical costs used in our study included out-of pocket payments by patients along with medical expenses paid by insurances, which is well appropriate for estimations from a societal perspective. We produced two range values of AF estimates with confidence intervals, based on a meta-analysis of RRs. Our calculation method had several strengths: we took account of multi-adjusted and age-adjusted estimates in our calculations, which produced the AF range values. And we combined this approach to meta-analyses to get more precise estimates of AFs based on available data in the literature. This method allowed us to encompass various high-quality RR estimates in our summary RRs, and at the same time to take account of a certain level of uncertainty regarding RRs, since age-adjusted and multi-adjusted estimates yielded different values.

Our summary RRs for CVD are consistent with those from the meta-analysis by Kivimaki et al. [28] providing a summary age and gender-adjusted RR of 1.45 (95% CI: 1.15-1.84) and a multi-adjusted RR of 1.16 (95% CI: 0.94-1.43). Our RR estimates regarding men are higher, but this difference could be explained by the fact that Kivimaki et al’s estimates included both men and women. Our results for MD are also consistent with those summarized in the meta-analysis by Stansfeld and Candy [30] (summary OR: 1.82, 95% CI: 1.06-3.10). Our estimates of attributable fractions for CVD mortality are more conservative than those reported by Nurminen & Karjalainen [19], who found estimates of 16% for men for the fractions of cardiovascular deaths attributable to job strain in Finland. Our AF estimates for MD are in line with those of LaMontagne et al. [77] who reported fractions of 13.2% for men and 17.2% for women attributable to job strain in Australia. Their fractions resulted from the summary OR estimates from Stansfeld and Candy’s meta-analysis [30]. Our results are higher than the costs of diseases attributable to job strain exposure in a previous study by Béjean et Sultan-Taïeb [25]. For the year 2000 in France, total costs amounted for 1.2-1.6 billion euros but included also musculoskeletal disorders. Differences can be explained by the fact that the fractions of MD for women were underestimated (4.8%) since they were based on a limited selection of OR estimates in the literature. The cost of depression attributable to job strain in Australia was estimated by LaMontagne et al. [26] at 730 million dollars AUD (approx.. 510 million euros) in 2007, given that 1.54 million persons suffer from depression in the Australian workforce. It is however difficult to compare these results with ours since categories of costs included in the estimations are different: job turnover and presenteeism costs are included in LaMontagne et al.’s study, while indirect costs related to premature death (suicide) are excluded.

Our results provide an evaluation at one point of time, allowing projections of the cost of job strain according to the evolution of working environments and the trend of prevalence of exposure to job strain. It also allows comparisons with other countries. Our summary estimates of RRs for CHD and MD could be used for AF calculations in other countries where the prevalence of exposure to job strain has been measured in the working population.

## Conclusions

This study on the economic burden of diseases attributable to job strain in France provides relevant insights for policy-makers when defining public health priorities for prevention policies. Diseases attributable to job strain and related costs are avoidable since effective intervention strategies to prevent job stress have been identified in several literature reviews [78-80]. Our results highlight potential economic implications of the development of such prevention policies.

## Competing interests

The authors declare that they have no competing interests.

## Authors’ contributions

HST conceived and conducted the study, directed the literature review of economic data, participated in the literature review of epidemiologic data, performed the statistical analysis of cost estimates, and wrote the manuscript. JFC performed the statistical analysis of summary RRs/ORs and prevalence of diseases, and contributed to the review of successive drafts of the manuscript. MM participated in the literature review of economic data. IN directed the literature review of epidemiologic data, and participated in study conception and manuscript writing. All authors read and approved the final manuscript.

## Pre-publication history

The pre-publication history for this paper can be accessed here:

http://www.biomedcentral.com/1471-2458/13/748/prepub

## References

[B1] World Health Organization Closing the gap in a generation : health equity through action on the social determinants of health. Final report of the Commission on Social Determinants of Health 2008 Geneva: World Health Organization 248

[B2] Cahalin LP Job strain and older workers: Can a reduction in job strain help to eliminate the social security drain? Work 2009 33 1 3 12 19597280 10.3233/WOR-2009-0838

[B3] Kalia M Assessing the economic impact of stress–the modern day hidden epidemic Metabolism 2002 51 6 Suppl 1 49 53 12040542 10.1053/meta.2002.33193

[B4] Shain M The role of the workplace in the production and containment of health costs: the case of stress-related disorders Int J Health Care Qual Assur Inc Leadersh Health Serv 1999 12 2–3 i vii 10537851 10.1108/13660759910266775

[B5] Liukkonen P Cartwright S Cooper C Kompier M, Cooper CL Costs and benefits of stress prevention in organisations Preventing stress, improving productivity: European case studies in the workplace 1999 London: Routledge 33 50

[B6] Koukoulaki T La prévention du stress au travail en Europe : aperçu des activités syndicales - obstacles et stratégies futures BTS Newsletter 2002 19–20 4 12

[B7] Brun JP Mental capital and wellbeing: Making the most of ourselves in the 21st century 2008 Quebec: Government Office for Science

[B8] Hoel H Sparks K Cooper CL The cost of violence/stress at work and the benefits of a violence/stress-free working environment. Report commissioned by the International Labour Organization (ILO) 2001 Geneva: University of Manchester Institute of Science and Technology

[B9] HSE (Health and Safety Executive) Workplace stress costs Great Britain in excess of £530 million. Press release: Health and Safety Executive C021:07(7/11/2007) [cited 2012] Available from: http://www.hse.gov.uk/press/2007/c07019.htm

[B10] Guthrie R Ciccarelli M Babic A Work-related stress in Australia: The effects of legislative interventions and the cost of treatment Int J Law Psychiatry 2010 33 2 101 115 Mar-Apr 10.1016/j.ijlp.2009.12.003 20116855

[B11] Biddle J Roberts K Rosenman KD Welch EM What percentage of workers with work-related illnesses receive workers’ compensation benefits? J Occup Environ Med 1998 40 4 325 331 10.1097/00043764-199804000-00006 9571523

[B12] Leigh JP Robbins JA Occupational disease and workers’ compensation: coverage, costs, and consequences Milbank Q 2004 82 4 689 721 10.1111/j.0887-378X.2004.00328.x 15595947 PMC2690178

[B13] Bonauto DK Smith CK Adams DA Fan ZJ Silverstein BA Foley MP Language preference and non-traumatic low back disorders in Washington State workers’ compensation Am J Ind Med 2010 53 2 204 215 10.1002/ajim.20740 19722197

[B14] Fayad R Nuwayhid I Tamim H Kassak K Khogali M Cost of work-related injuries in insured workplaces in Lebanon Bull World Health Organ 2003 81 7 509 516 12973643 PMC2572495

[B15] Fingerhut M Driscoll T Nelson DI Concha-Barrientos M Punnett L Pruss-Ustin A Contribution of occupational risk factors to the global burden of disease - a summary of findings Scand J Work Environ Health 2005 1 suppl 59 61

[B16] Nelson DI Concha-Barrientos M Driscoll T Steenland K Fingerhut M Punnett L The global burden of selected occupational diseases and injury risks: Methodology and summary Am J Ind Med 2005 48 6 400 418 10.1002/ajim.20211 16299700

[B17] Driscoll T Takala J Steenland K Corvalan C Fingerhut M Review of estimates of the global burden of injury and illness due to occupational exposures Am J Ind Med 2005 48 6 491 502 10.1002/ajim.20194 16299705

[B18] Steenland K Burnett C Lalich N Ward E Hurrell J Dying for work: The magnitude of US mortality from selected causes of death associated with occupation Am J Ind Med 2003 43 5 461 482 10.1002/ajim.10216 12704620

[B19] Nurminen M Karjalainen A Epidemiologic estimate of the proportion of fatalities related to occupational factors in Finland Scand J Work Environ Health 2001 27 3 161 213 10.5271/sjweh.605 11444413

[B20] t’Mannetje A Pearce N Quantitative estimates of work-related death, disease and injury in New Zealand Scand J Work Environ Health 2005 31 4 266 276 Aug 10.5271/sjweh.882 16161709

[B21] Leigh JP Markowitz SB Fahs M Shin C Landrigan PJ Occupational injury and illness in the United States. Estimates of costs, morbidity, and mortality Arch Intern Med 1997 157 14 1557 1568 10.1001/archinte.1997.00440350063006 9236557

[B22] Leigh JP Cone JE Harrison R Costs of occupational injuries and illnesses in California Prev Med 2001 32 5 393 406 10.1006/pmed.2001.0841 11330988

[B23] Leigh JP Economic burden of occupational injury and illness in the United States Milbank Q 2011 89 4 728 772 10.1111/j.1468-0009.2011.00648.x 22188353 PMC3250639

[B24] Levi L Lunde Jensen P A model for assessing the costs of stressors at national level: socio-economic costs of work stress in two EU member states 1996 Dublin: European Foundation for the Improvement of Living and Working Conditions

[B25] Bejean S Sultan-Taïeb H Modeling the economic burden of diseases imputable to stress at work Eur J Health Econ 2005 50 1 16 23 15452742 10.1007/s10198-004-0251-4

[B26] LaMontagne AD Sanderson K Cocker F Estimating the economic benefits of eliminating job strain as a risk factor for depression 2010 Carlton, Australia: Victorian Health Promotion Foundation (VicHealth) 10.1097/JOM.0000000000000908 28045792

[B27] Leigh JP Expanding research on the economics of occupational health Scand J Work Environ Health 2006 32 1 1 4 10.5271/sjweh.969 16539165

[B28] Kivimäki M Virtanen M Elovainio M Kouvonen A Vaananen A Vahtera J Work stress in the etiology of coronary heart disease–a meta-analysis Scand J Work Environ Health 2006 32 6 431 442 10.5271/sjweh.1049 17173200

[B29] Belkic KL Landsbergis PA Schnall PL Baker D Is job strain a major source of cardiovascular disease risk? Scand J Work Environ Health 2004 30 2 85 128 10.5271/sjweh.769 15127782

[B30] Stansfeld S Candy B Psychosocial work environment and mental health–a meta-analytic review Scand J Work Environ Health 2006 32 6 443 462 10.5271/sjweh.1050 17173201

[B31] Bonde JP Psychosocial factors at work and risk of depression: a systematic review of the epidemiological evidence Occup Environ Med 2008 65 7 438 445 10.1136/oem.2007.038430 18417557

[B32] Netterstrom B Conrad N Bech P Fink P Olsen O Rugulies R The relation between work-related psychosocial factors and the development of depression Epidemiol Rev 2008 30 118 132 10.1093/epirev/mxn004 18587142

[B33] Bongers PM Ijmker S Van den Heuvel S Blatter BM Epidemiology of work related neck and upper limb problems: psychosocial and personal risk factors (part I) and effective interventions from a bio behavioural perspective (part II) J Occup Rehabil 2006 16 3 279 302 16850279 10.1007/s10926-006-9044-1

[B34] Sultan-Taieb H Lejeune C Drummond A Niedhammer I Fractions of cardiovascular diseases, mental disorders, and musculoskeletal disorders attributable to job strain Int Arch Occup Environ Health 2011 84 8 911 925 10.1007/s00420-011-0633-8 21461767

[B35] Karasek R Brisson C Kawakami N Houtman I Bongers P Amick B The Job Content Questionnaire (JCQ): an instrument for internationally comparative assessments of psychosocial job characteristics J Occup Health Psych 1998 3 4 322 355 10.1037//1076-8998.3.4.322 9805280

[B36] Drummond M Cost-of-illness studies: a major headache? Pharmacoeconomics 1992 2 1 1 4 10.2165/00019053-199202010-00001 10146974

[B37] Drummond M Sculpher M Torrance G Methods for the economic evaluation of health care programmes 2005 3 Oxford: Oxford University Press 379 xv

[B38] Gold MR Siegel JE Russell LB Weinstein MC Cost-effectiveness in health and medicine 1996 New York: Oxford University Press

[B39] Byford S Raftery J Perspectives in economic evaluation Bmj 1998 316 7143 1529 1530 9582152 10.1136/bmj.316.7143.1529 PMC1113167

[B40] Torrance G Blaker D Detsky A Kennedy W Schubert F Menon D Canadian Guidelines for Economic Evaluation of Pharmaceuticals Pharmacoeconomics 1996 9 6 535 559 10.2165/00019053-199609060-00008 10160481

[B41] Levin ML The occurrence of lung cancer in man Acta Unio Int Contra Cancrum 1953 9 3 531 541 13124110

[B42] Niedhammer I Chastang JF Levy D David S Degioanni S Theorell T Study of the validity of a job-exposure matrix for psychosocial work factors: results from the national French SUMER survey Int Arch Occup Environ Health 2008 82 1 87 97 10.1007/s00420-008-0311-7 18327603

[B43] Bosma H Peter R Siegrist J Marmot M Two alternative job stress models and the risk of coronary heart disease Am J Public Health 1998 88 1 68 74 10.2105/AJPH.88.1.68 9584036 PMC1508386

[B44] De Bacquer D Pelfrene E Clays E Mak R Moreau M de Smet P Perceived job stress and incidence of coronary events: 3-year follow-up of the Belgian Job Stress Project cohort Am J Epidemiol 2005 161 5 434 441 10.1093/aje/kwi040 15718479

[B45] Kivimäki M Ferrie JE Brunner E Head J Shipley MJ Vahtera J Justice at work and reduced risk of coronary heart disease among employees: the Whitehall 2 Study Arch Intern Med 2005 165 19 2245 2251 10.1001/archinte.165.19.2245 16246990

[B46] Kivimäki M Theorell T Westerlund H Vahtera J Alfredsson L Job strain and ischaemic disease: does the inclusion of older employees in the cohort dilute the association? The WOLF Stockholm Study J Epidemiol Community Health 2008 62 4 372 374 10.1136/jech.2007.063578 18339833

[B47] Kornitzer M DeSmet P Sans S Dramaix M Boulenguez C DeBacker G Job stress and major coronary events: results from the Job Stress, Absenteeism and Coronary Heart Disease in Europe study Eur J Cardiov Prev R 2006 13 5 695 704 Oct 10.1097/01.hjr.0000221865.19415.e9 17001207

[B48] Lee S Colditz G Berkman L Kawachi I A prospective study of job strain and coronary heart disease in US women Int J Epidemiol 2002 31 6 1147 1154 10.1093/ije/31.6.1147 12540714

[B49] Netterstrom B Kristensen TS Sjol A Psychological job demands increase the risk of ischaemic heart disease: a 14-year cohort study of employed Danish men Eur J Cardiov Prev R 2006 13 3 414 420 10.1097/00149831-200606000-00018 16926672

[B50] Orth-Gomer K Wamala SP Horsten M Schenck-Gustafsson K Schneiderman N Mittleman MA Marital stress worsens prognosis in women with coronary heart disease: The Stockholm Female Coronary Risk Study Jama 2000 284 23 3008 3014 10.1001/jama.284.23.3008 11122587

[B51] Uchiyama S Kurasawa T Sekizawa T Nakatsuka H Job strain and risk of cardiovascular events in treated hypertensive Japanese workers: hypertension follow-up group study J Occup Health 2005 47 2 102 111 10.1539/joh.47.102 15824474

[B52] Bildt C Michelsen H Gender differences in the effects from working conditions on mental health: a 4-year follow-up Int Arch Occup Environ Health 2002 75 4 252 258 10.1007/s00420-001-0299-8 11981659

[B53] Bourbonnais R Comeau M Vezina M Job strain and evolution of mental health among nurses J Occup Health Psychol 1999 4 2 95 107 10212863 10.1037//1076-8998.4.2.95

[B54] Clays E De Bacquer D Leynen F Kornitzer M Kittel F De Backer G Job stress and depression symptoms in middle-aged workers–prospective results from the Belstress study Scand J Work Environ Health 2007 33 4 252 259 10.5271/sjweh.1140 17717616

[B55] Niedhammer I Goldberg M Leclerc A Bugel I David S Psychosocial factors at work and subsequent depressive symptoms in the Gazel cohort Scand J Work Environ Health 1998 24 3 197 205 10.5271/sjweh.299 9710372

[B56] Shields M Long working hours and health Health Rep 1999 11 2 33 48 10618741

[B57] Shields M Stress and depression in the employed population Health Rep 2006 17 4 11 29 17111591

[B58] Higgins JP Thompson SG Deeks JJ Altman DG Measuring inconsistency in meta-analyses Bmj 2003 327 7414 557 560 10.1136/bmj.327.7414.557 12958120 PMC192859

[B59] Niedhammer I Chastang JF Gendrey L David S Degioanni S Propriétés psychométriques de la version française des échelles de la demande psychologique, de la latitude décisionnelle et du soutien social du “Job Content Questionnaire” de Karasek: Résultats de l’enquête nationale SUMER Sante Publique 2006 18 3 413 427 10.3917/spub.063.0413 17094683

[B60] Niedhammer I Chastang JF David S Importance of psychosocial work factors on general health outcomes in the national French SUMER survey Occupational Medicine (London) 2008 58 1 15 24 10.1093/occmed/kqm115 17965447

[B61] Niedhammer I Chastang JF David S Kelleher C The contribution of occupational factors to social inequalities in health: findings from the national French SUMER survey Soc Sci Med 2008 67 11 1870 1881 10.1016/j.socscimed.2008.09.007 18851892

[B62] Niedhammer I Chastang J-F Levy D David S Degioanni S Exposition aux facteurs psychosociaux au travail du modèle de Karasek: étude méthodologique à l’aide de l’enquête nationale SUMER Travailler 2007 17 47 70 10.3917/trav.017.0047

[B63] CEPIDC (Centre d’Épidémiologie sur les Causes Médicales de Décès) Interrogation des données sur les causes de décès de 1979 à 2010 2003 [cited 2012]. Available from: http://www.cepidc.inserm.fr/inserm/html/index2.htm

[B64] Isometsa E Henriksson M Marttunen M Heikkinen M Aro H Kuoppasalmi K Mental disorders in young and middle aged men who commit suicide Bmj 1995 310 6991 1366 1367 10.1136/bmj.310.6991.1366 7787539 PMC2549746

[B65] Bertolote JM Fleischmann A De Leo D Wasserman D Suicide and mental disorders: do we know enough? The British journal of psychiatry 2003 183 382 383 10.1192/bjp.183.5.382 14594911

[B66] Michel G Aquaviva E Aubron V Purper-Ouakil D Beck F, Guilbert P, Gautier A Suicides: mieux comprendre pour prévenir avec plus d’efficacité Baromètre santé 2005 2007 Saint-Denis: Institut National de Prévention et d’Education pour la Santé, (INPES) p. 487 506

[B67] Lepine JP Gasquet I Kovess V Arbabzadeh-Bouchez S Negre-Pages L Nachbaur G [Prevalence and comorbidity of psychiatric disorders in the French general population] Encéphale 2005 31 2 182 194 Mar-Apr 15959445 10.1016/s0013-7006(05)82385-1

[B68] Rice DP Estimating the cost of illness Am J Public Health 1967 57 3 424 440 10.2105/AJPH.57.3.424 PMC1227175 6066903

[B69] Paris V Renaud T Sermet C Des comptes de la santé par pathologie : un prototype pour l’année 1998 2003 Paris: Centre de recherche d’étude et de documentation en économie de la santé (CREDES) 14506447

[B70] Fenina A Geoffroy Y Minc C Renaud T Sarlon E Sermet C Les dépenses de prévention et les dépenses de soins par pathologie en France Questions d’Économie de la Santé 2006 111 1 823945431

[B71] Eco-Santé France Bases Eco-Santé France: IRDES (Institut de Recherche et Documentation en Économie de la Santé) 2003 [cited 2012]. Available from: http://www.irdes.fr/EcoSante/France.htm

[B72] Morvan Y Prieto A Briffault X Blanchet A Dardennes R Rouillon F Beck F, Guilbert P, Gautier A La dépression: prévalence, facteurs associés et consommation de soins Baromètre santé 2005 2007 Saint-Denis: Institut National de Prévention et d’Education pour la Santé (INPES) 487 506

[B73] De Peretti C Chin F Tuppin P Danchin D Personnes hospitalisées pour infarctus du myocarde en France: tendances 2002–2008 Bulletin Epidémiologique Hebdomadaire 2012 41 459 465

[B74] Pérel C Chin F Tuppin P Danchin D Alla F Juillière Y Taux de patients hospitalisés pour insuffisance cardiaque en 2008 et évolutions en 2002–2008, France Bulletin Epidémiologique Hebdomadaire 2012 41 466 470

[B75] Siegrist J Starke D Chandola T Godin I Marmot M Niedhammer I The measurement of effort-reward imbalance at work: European comparisons Soc Sci Med 2004 58 8 1483 1499 10.1016/S0277-9536(03)00351-4 14759692

[B76] LaMontagne AD Keegel T Louie AM AO Job stress as a preventable upstream determinant of common mental disorders: A review for practitioners and policy-makers Advances in Mental Health 2010 9 1 17 35 10.5172/jamh.9.1.17

[B77] LaMontagne AD Keegel T Vallance D Ostry A Wolfe R Job strain - attributable depression in a sample of working Australians: assessing the contribution to health inequalities BMC Publ Health 2008 8 181 10.1186/1471-2458-8-181 PMC2416448 18505559

[B78] LaMontagne AD Keegel T Louie AM Ostry A Landsbergis PA A systematic review of the job-stress intervention evaluation literature, 1990–2005 Int J Occup Environ Health 2007 13 3 268 280 Jul-Sep 17915541 10.1179/oeh.2007.13.3.268

[B79] Egan M Bambra C Thomas S Petticrew M Whitehead M Thomson H The psychosocial and health effects of workplace reorganisation. 1. A systematic review of organisational-level interventions that aim to increase employee control J Epidemiol Community Health 2007 61 11 945 954 10.1136/jech.2006.054965 17933951 PMC2465601

[B80] Bambra C Egan M Thomas S Petticrew M Whitehead M The psychosocial and health effects of workplace reorganisation. 2. A systematic review of task restructuring interventions J Epidemiol Community Health 2007 61 12 1028 1037 10.1136/jech.2006.054999 18000123 PMC2465678

